# Machine Learning-based Prognostic Model for Brain Metastasis Patients: Insights from Blood Test Analysis

**DOI:** 10.7150/jca.103847

**Published:** 2025-01-01

**Authors:** Ruidan Li, Zheran Liu, Zhigong Wei, Rendong Huang, Yiyan Pei, Jing Yang, Zijian Qin, Huilin Li, Fang Fang, Xingchen Peng

**Affiliations:** 1 Department of Biotherapy, Cancer Center, West China Hospital, Sichuan University, Chengdu, Sichuan, China.; 2 Hangzhou Linan Guorui Health Industry Investment Co.,Ltd, Hangzhou, Zhejiang, China.; 3 International Center for Aging and Cancer, Hainan Medical University, Haikou, China.; 4 Department of Neurosurgery, West China Hospital, Sichuan University, Chengdu, Sichuan, China.

**Keywords:** machine learning, prognostic model, brain metastasis, hematological parameter

## Abstract

**Purpose:** Brain metastases, affecting 30% of solid tumor patients, have a substantial impact on clinical outcomes. Developing a clinically feasible and precise prognostic model is crucial for personalized and comprehensive treatment.

**Methods:** Parameters from blood test were collected from brain metastases patients, and were used to construct the four models, including univariate Cox regression, stepwise regression, LASSO regression, and random survival forest (RSF). Model-HP (based RSF), identified as the best-performing, was chosen. Model-GPAH was formed by merging Model-HP risk scores and GPA (Graded Prognostic Assessment). AUC, IDI, and cNRI were used to evaluate different models.

**Results:** A cohort of 1,385 patients was included, with 970 patients assigned to the training cohort and 415 patients were to the validation cohort. Compared to the other models, the Model-HP built on the RSF demonstrated superior performance (compared with RSF: AUC = 0.71 [0.66, 0.77], Univariate Cox regression: AUC = 0.65 [0.59, 0.71], P = 0.011; Stepwise regression: AUC = 0.63 [0.57, 0.69], P = 0.001; LASSO regression: AUC = 0.64 [0.58, 0.70], P < 0.001). Compared with Model-HP and GPA, Model-GPAH significantly enhanced the performance of prognosis prediction (compared with Model-GPAH: AUC = 0.70 [0.67, 0.73], GPA: AUC = 0.61 [0.57, 0.64], P = 0.001; Model-HP: AUC = 0.67 [0.64, 0.70], P < 0.001). Model-GPAH performed favorably across patients receiving diverse treatments.

**Conclusions:** Integrating hematological parameters into the GPA model significantly enhanced prognostic prediction for brain metastasis patients, highlighting blood tests' crucial role in identifying biomarkers for outcomes.

## Introduction

Approximately 30% of solid tumor patients may experience brain metastases, where lung cancer, breast cancer, and melanoma are commonly associated, accounting for 67%-80% of cases 1, 2. Brain metastases is closely related to adverse clinical outcomes and poor overall survival 3. The treatment for brain metastases patients undergoes a significant shift compared to the pre-metastatic stage. Adopting a multidisciplinary strategy is crucial to customize treatments for brain metastases patients 3. Clinically feasible and accurate prognostic stratification may contribute to providing a reliable basis for personalized treatment decisions for these patients.

The prognostic scoring systems of patients with brain metastasis have consistently evolved over the years 4-10. Differing from other prognostic scoring systems, the Graded Prognostic Assessment (GPA) is independent of primary tumor control, a subjective variable that is often challenging to consistently assess 9. In the GPA, a cumulative score is assigned to each patient based on four weighted variables, including age, Karnofsky Performance Status (KPS), the number of brain metastases, and the presence of extracranial metastases 9. In recent years, new prognostic criteria, extending the foundation laid by GPA, have incorporated additional biomarkers, like the Epidermal Growth Factor Receptor mutation or Anaplastic Lymphoma Kinase rearrangement status. However, these prognostic factors are only applicable to patients with non-small cell lung cancers that have brain metastases 11, 12. Developing a new prognostic scoring system applicable to a wider range of tumors is essential for offering practical estimates of survival.

Recently, machine learning (ML) has exhibited remarkable efficacy in various medical applications, including diagnosis, clinical characterization, risk categorization, and predicting treatment responses 13. In this study, we employed two ML methods, including Least Absolute Shrinkage and Selection Operator (LASSO) and Random Survival Forest (RSF), to screen parameters from the blood test related to the prognosis of patients with brain metastases. Following screening, the identified key parameters, in collaboration with the GPA, establish an effective prognostic model for forecasting the prognosis of patients with brain metastases.

## Methods

### Patient cohort and data collection

From December 2013 to August 2021, we retrospectively collected data on patients diagnosed and treated for brain metastasis at West China Hospital of Sichuan University. The details of this cohort have been described previously 14.

In this study, the demographic and clinical information were meticulously collected. Baseline characteristics, detailed clinical presentations, imaging findings, and results from blood tests were included. Notably, the KPS of each patient was rigorously evaluated by two independent oncologists, namely Dr. Xingchen Peng and Dr. Zhigong Wei, ensuring an unbiased and thorough assessment. Common blood test (hematological parameters from blood test were available in over 75% patients in our cohort) were systematically extracted using the unique hospital registration ID of each patient.

The criteria for patient inclusion and exclusion were as follows. Inclusion criteria: (1) The primary solid tumor was diagnosed. (2) Brain metastasis was confirmed at the initial diagnosis or upon relapse through imaging examinations. Exclusive criteria: (1) patients without available blood test results. (2) patients with unknown survival status.

### Outcome

The primary outcome was overall survival (OS), defined as the duration from the initial brain metastasis diagnosis to either death or the study's conclusion on September 30, 2020, whichever occurred first. Survival status and death dates were queried through the Sichuan Province Household Registration Administration System databases.

### The construction of Model-HP

**Figure 1:**
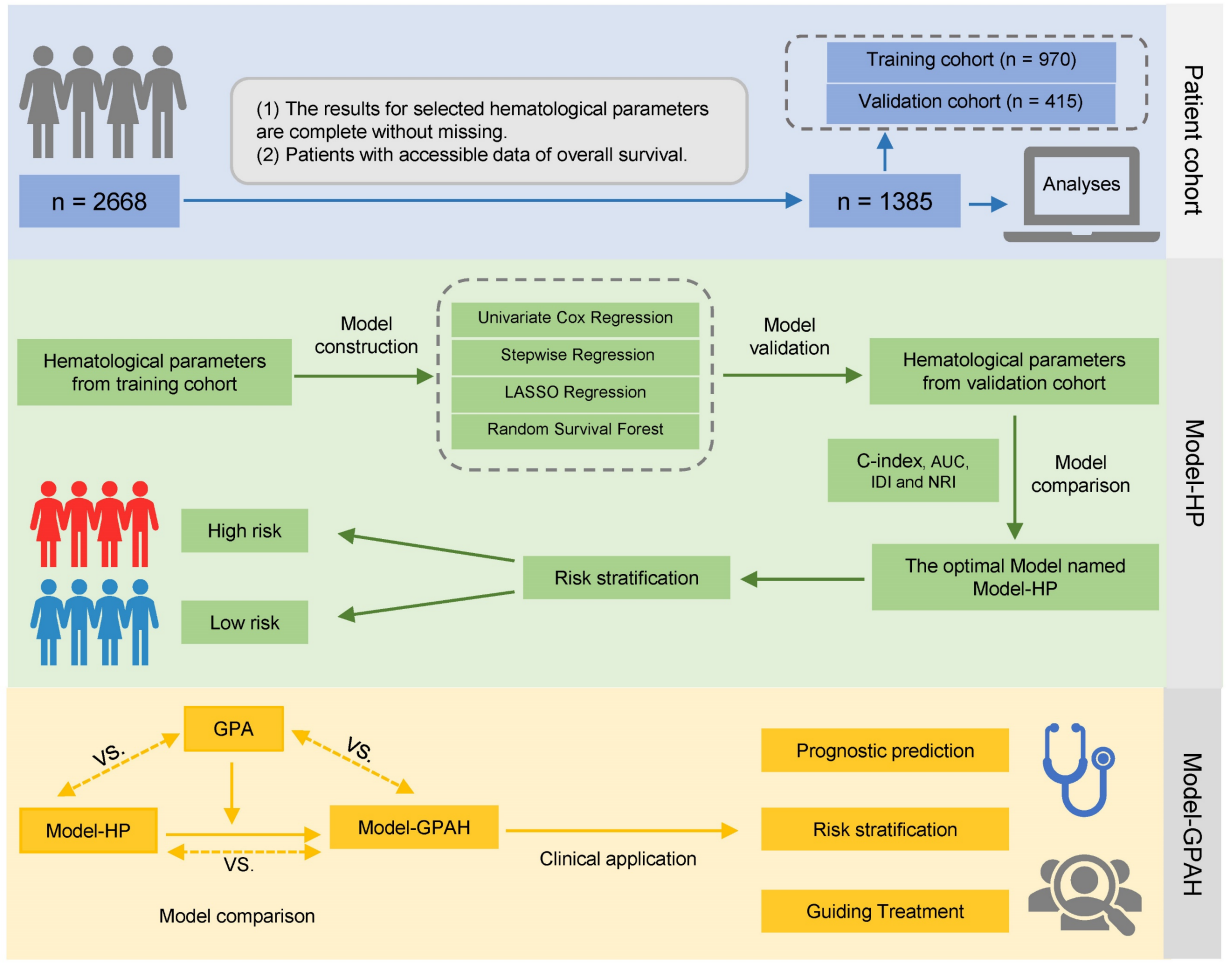
The design of the study.

Sixty-three hematological parameters were eventually selected (Table S1). A total of 1385 patients were allocated to the training and validation cohorts at a fixed ratio of 7:3 randomly. Four models were established to screen hematological parameters, leading to the construction of the effective Model-HP (Fig. 1). The first model was a multivariate Cox model based on hematological parameters which significantly related to OS in the univariate Cox model (significant threshold: p < 0.01). The second model was a stepwise multivariate Cox model based on hematological parameters which screened by stepwise Cox regression in the forward conditional method and the lowest Akaike Information Criterion (AIC) was the indicator to select the variables. The third model was a LASSO regression model. LASSO regression was used to select variables and Cox regression was applied to calculate the estimates. LASSO regression models were used to eliminate unimportant variables by penalizing the regression coefficients, shrinking them toward zero, and the degree of shrinkage depended on an additional parameter, λ. A 10-fold cross-validation was performed to determine the optimal value for λ based on the minimum deviance criteria.

The fourth model was RSF. The variable importance (VIMP) was obtained to assess the predictive capability of predictor variables. A positive VIMP value signified a predictive effect and the higher VIMP value indicated the stronger predictive ability. The top 25 important variables were selected, and then the RSF model was rebuilt.

The accuracy and concordance of four models were compared with each other and with a reference model (univariate Cox model constructed by 63 hematological parameters) in a validation cohort using Area Under the Receiver Operating Characteristic Curve (AUC), continuous Net Reclassification Improvement (cNRI), and Integrated Discrimination Improvement (IDI). Integrating the above indicators, the optimal hematological model was selected and named Model-HP (model based on hematological parameters).

### The construction of Model-GPAH

Based on Model-HP, indicators from GPA, such as age, KPS, brain metastases count, and the status of extracranial metastases, were incorporated into Model-HP, forming Model-GPAH (GPA with hematological parameters). The missing values for these GPA-related variable were imputed using random forest imputation. These three models were compared using AUC, IDI, and cNRI with the same procedure in the construction of Model-HP part. A nomogram was used to visualize the prognostic model. The calibration plots were employed to exhibit the accordance between predicted survival and actual survival.

### Subgroup analysis

For patients who received different treatments, we systematically analyzed their outcomes by repeatedly assessing OS and recalculating the performance for Model-GAPH.

### Sensitivity analysis

The data of patients without imputation was incorporated into Model-GPAH for reanalysis. This enabled a thorough examination of the model's performance across the diverse cohort, thereby bolstering the robustness and applicability of the findings.

### Statistical analyses

Continuous variables were reported with the mean value accompanied by the standard deviation (SD), while categorical data were presented as the count and respective percentages for each group. Group differences were examined by employing T-tests or chi-square tests.

The predicted scores yield by Model-HP and Model-GPAH were defined as the risk score of the corresponding models. According to the cut-off value, patients were divided into high risk score and low risk score.

To illustrate the time-to-event data, Kaplan-Meier curves were employed, while log-rank tests were conducted to evaluate the differences in overall survival across various risk group.

All analyses were performed with R 4.3.2. A 2-sided p<0.05 was considered statistically significant.

## Results

### Population characteristics

Based on the criteria for variable selection, a total of 63 hematological parameters were included for subsequent analysis. A detailed list of these selected variables can be found in Table S1. Following inclusion and exclusion criteria, a cohort of 1,385 patients was included. For these included participants, lung cancer emerged as the predominant primary tumor, constituting 78% (1,080 cases). Extracranial metastases were observed in 65.1% (901 patients), and 35.2% (487 patients) presented with more than three brain metastases. The included participants were randomly divided in a 7:3 ratio, with 970 patients in the training cohort and 415 patients in the validation cohort. The population characteristics was provided in Table 1.

### The construction and selection of Model-HP

The reference model was built by incorporated the 63 hematological parameters in one multivariate Cox model. The significant hematological parameters in the reference model were listed in Table S2.

Four models (i.e., univariate Cox regression, stepwise regression, LASSO regression and RSF) were constructed according to the established rules. In the multivariate Cox model that adjusted all of the significant hematological parameters in univariate Cox model, only LDH showed a significant association with OS (Fig. S1). In the stepwise multivariate Cox model, LDH was excluded from the model while the absolute immature granulocytes, AST/ALT, and platelet count showed significant association with OS (Fig. S2). In the Lasso-Cox model, the iterative analysis involved the implementation of a 10-fold cross-validation method, resulting in a model with exceptional performance at the minimized λ (0.038, Log λ=-1.42). Twenty variables were selected into the Cox model; the details were listed in Figure S3. For the RFS model, the important variables selected by the RFS model were listed in Figure S4 and LDH has been assessed as the most important variable.

**Figure 2:**
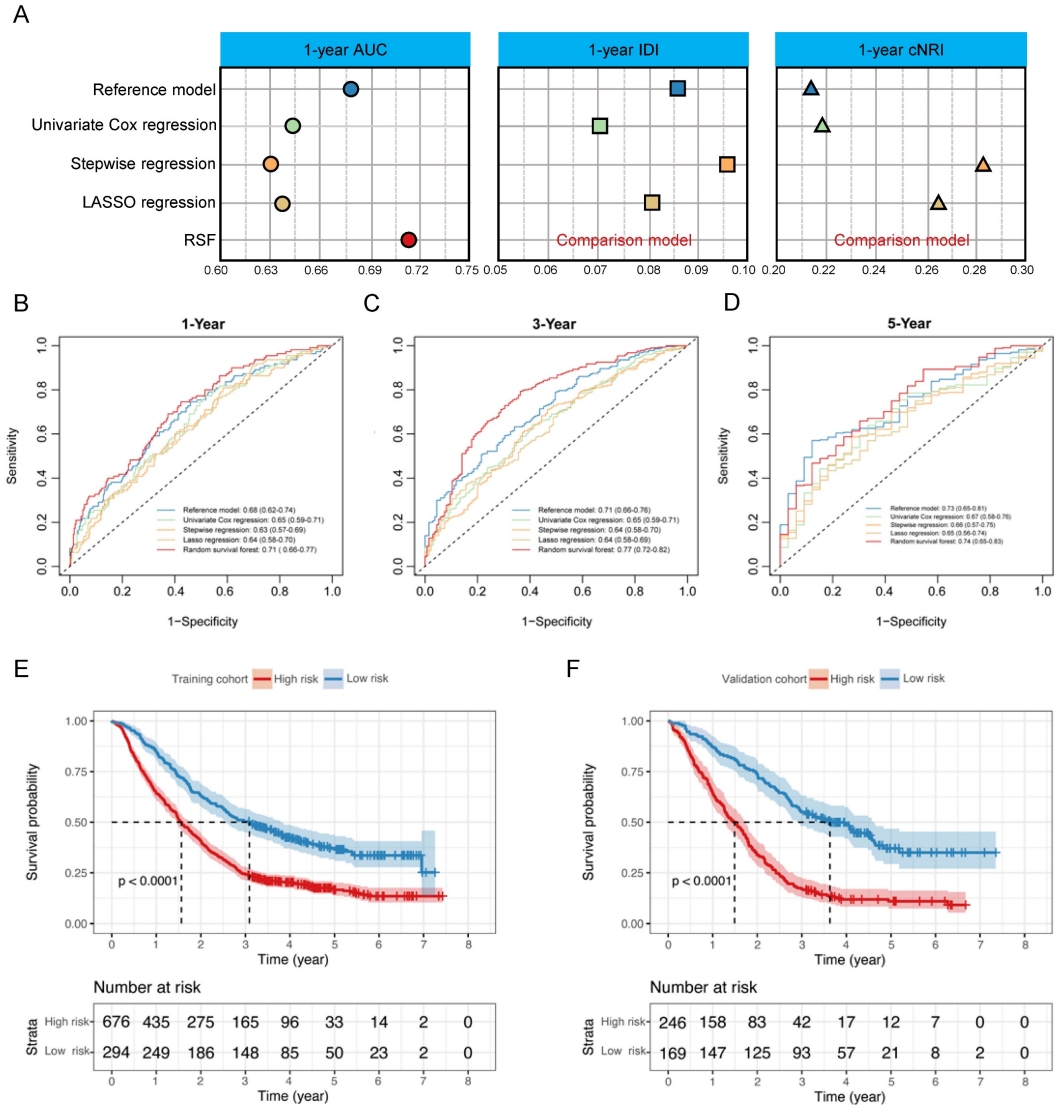
** The construction of Model-HP.** (A)the comparison of AUC, IDI and NRI between the reference model, Univariate Cox regression, Stepwise regression, Lasso regression, and RSF. The 1-year (B), 3-year (C), and 5-year (D) ROC curves for the reference model, Univariate Cox regression, Stepwise regression, Lasso regression, and RSF. Kaplan-Meier curves of OS for patients in different risk levels in the training cohort (E) and validation cohort (F). GPA, Graded Prognostic Assessment; Model-HP, model based on hematological parameters; RSF, random survival forest; ROC, Receiver Operating Characteristic.

In the model comparison part, we decided that the RFS model was the best-performing model compared with the other 3 models according to the comparison of AUC, IDI and NRI (Fig. 2A-D and Table S3). Thus, it was designated as Model-HP. Risk scores in Model-HP were calculated for the patients, and an optimal cutoff of 0.79 was determined in the training cohort. Subsequently, patients were stratified into high-risk and low-risk groups based on this threshold. Survival analysis revealed that, compared to the low-risk group, the high-risk group showed significantly inferior OS in both the training cohort (P < 0.0001, Fig. 2E) and the validation cohort (P < 0.0001, Fig. 2F).

### The construction of Model-GPAH

Utilizing Model-HP as a foundation, parameters derived from the GPA (i.e., age, KPS, brain metastases count, and the status of extracranial metastases) were integrated into Model-HP, thereby giving rise to Model-GPAH.

**Figure 3:**
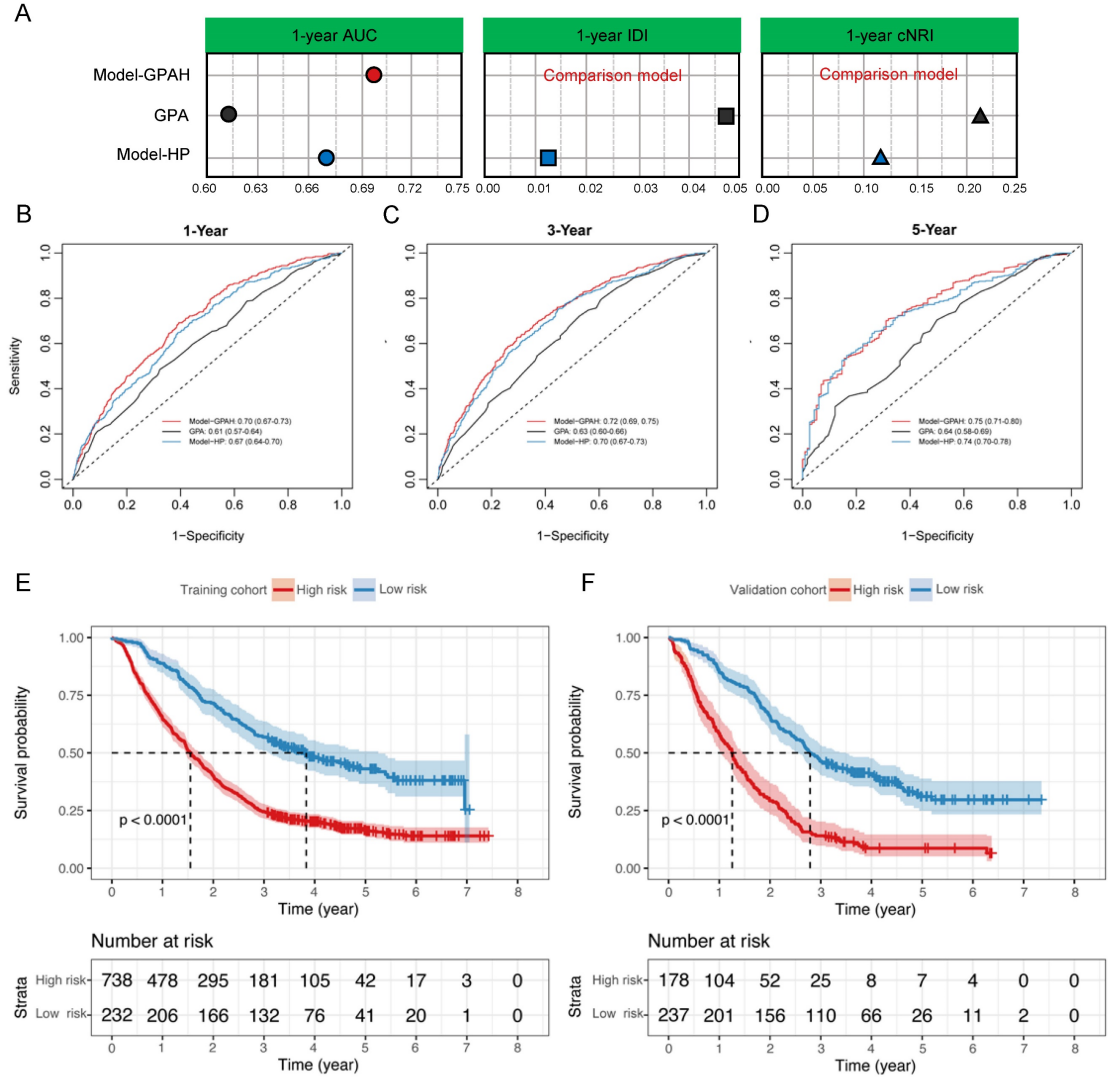
** The construction of Model-GPAH.** (A) the comparison of AUC, IDI and NRI between Model-HP, GPA, and Model-GPAH. the 1-year (B), 3-year (C), and 5-year (D) ROC curves for the Model-HP, GPA, and Model-GPAH. Kaplan-Meier curves of OS for patients in different risk levels in the training cohort (D) and validation cohort (E). GPA, Graded Prognostic Assessment; Model-HP, model based on hematological parameters; Model-GPAH, model based on GPA and hematological parameters; ROC, Receiver Operating Characteristic.

**Figure 4:**
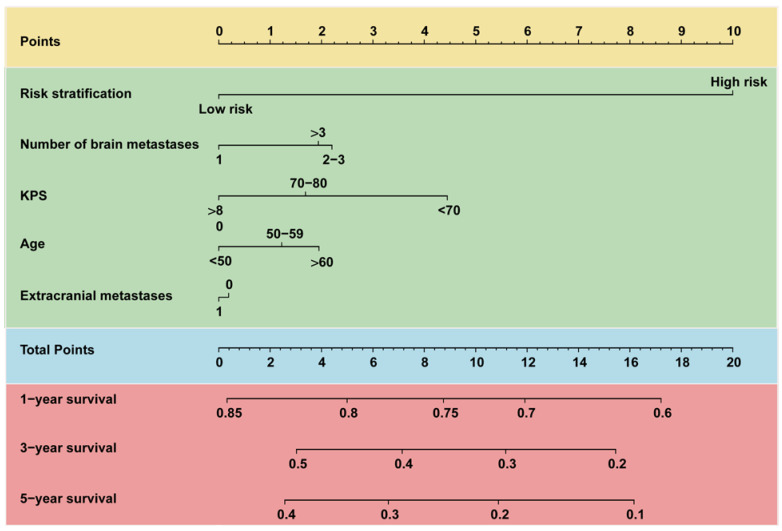
** Nomogram for predicting 1-, 3-, and 5-year OS of patients with brain metastasis.** For each patient, five lines are drawn upward to determine the points received from the predictors in the nomogram. The sum of these points is located on the “Total Points” axis. Besides, three lines are drawn downward to determine the possibility of 1-, 3-, and 5-year OS. OS, overall survival.

In the comparison among GPA, Model-HP, and Model-GPAH, the results showed that Model-GPAH significantly outperformed GPA (Fig. 3A-D and Table S4). This suggested that the inclusion of hematological parameters significantly enhanced the model performance. However, compared to Model-HP, Model-GPAH did not demonstrate a superior advantage in assessments. This might be attributed to the inherently strong performance of Model-HP. Based on Model-GAPH, a Nomogram was established (Fig. 4). The calibration plot demonstrated high accuracy in predicting the probability, aligning closely with both actual and predicted probabilities (Fig. S5).

Risk scores were calculated for patients in Model-GAPH, and an optimal cutoff of 0.53 was identified in the training cohort to stratify patients into high-risk and low-risk groups. The results revealed that the high-risk group exhibited significantly poorer OS compared to the low-risk group in both the training cohort (P < 0.0001, Fig. 3E) and the validation cohort (P < 0.0001, Fig. 3F).

### Subgroup analysis

**Figure 5:**
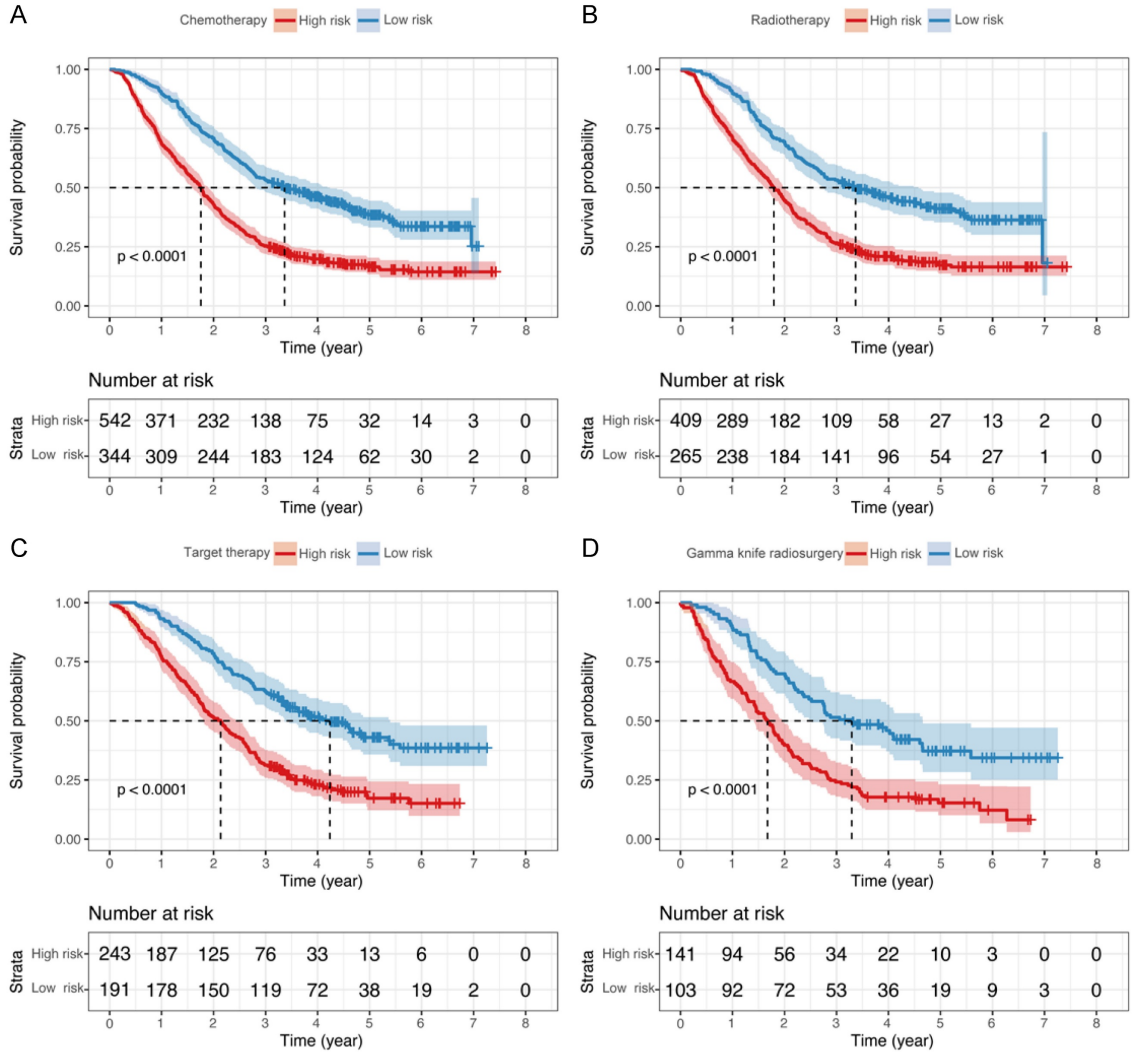
** Kaplan-Meier curves of OS for patients who underwent different treatments.** The survival of the low- and high-risk groups in patients with chemotherapy (A), radiotherapy (B), Target therapy (C), and gamma knife radiosurgery (D). OS, overall survival.

In subgroup analyses, we independently reanalyzed patients that underwent chemotherapy, targeted therapy, radiotherapy, and gamma knife radiosurgery in Model-GPAH. The results showed outstanding performance by Model-GPAH in the chemotherapy, targeted therapy, radiotherapy, and gamma knife radiosurgery subgroups. Across these four subgroups, individuals identified as high-risk by Model-GPAH consistently demonstrated significantly worse prognosis than low-risk patients (Fig. 5).

### Sensitivity analysis

To further validate the model's applicability in diverse populations, the data of patients without imputation (Table S5) was incorporated into Model-GPAH for reanalysis. The results indicated that the patients classified as high-risk demonstrated markedly poor OS in comparison to the low-risk group. (P < 0.0001, Fig. S6).

## Discussion

In this comprehensive retrospective cohort study involving patients with brain metastases from solid tumors, we utilized four distinct algorithms to construct and selectively identify an optimal hematologic parameter model, designated as Model-HP, which was employed for prognostic predictions in brain metastases patients. The integration of Model-HP risk score and the GPA resulted in a composite model referred to as Model-GPAH. Compared to GPA, Model-GPAH significantly enhanced the prognostic capability for patients with brain metastases and was applicable to individuals receiving various treatments.

Hematological analysis stands out as a frequently employed diagnostic method in clinical practice. Its cost-effectiveness not only made it the preferred choice for patients but also facilitated researchers in obtaining the necessary test results more readily. In numerous past studies, researchers have successfully identified key biomarkers associated with the prognosis of tumors like colorectal cancer, breast cancer and lung cancer through the analysis of routine hematology examinations 15-17. However, the significance of hematological parameters has not yet been recognized in patients with brain metastases. In this research, we employed various methodologies to construct a model, conducting a comprehensive comparison to identify an optimal model built by RSF centered on hematological parameters. Compared to prior studies, our model was applicable to brain metastases patients resulting from various solid tumors, showcasing a superior AUC value 18, 19. This suggested that our model held broad applicability and remarkable discriminatory capability in predicting the prognosis of brain metastases patients.

The hematological parameters identified in this study have been discussed in several previous studies. Lactate dehydrogenase (LDH) and Hydroxybutyrate dehydrogenase (HBDH) have been recognized as crucial prognostic indicators in cancer patients 14, 20. The Albumin-to-Alkaline Phosphatase Ratio (AAPR), as proposed by numerous studies, has been substantiated as a critical prognostic determinant for patients with various solid tumors. A diminished pretreatment AAPR was notably related to unfavorable clinical outcomes 21, 22. Similar to this study, earlier research findings suggested there was a potential association between coagulation-related biomarkers and the prognosis of cancer patients. Notably, there was a significant association between fibrinogen (FIB) and the prognosis of patients with esophageal and prostate cancer 23, 24. Additionally, thrombin time (TT) showed a significant correlation with the survival outcome of breast cancer patients 25. Furthermore, the RSF model contributed to identifying indicators, such as High-density lipoprotein (HDL) and Triglyceride, linked to lipid metabolism. This suggested that disturbances in lipid metabolism might contribute to tumor progression 26, 27. In addition to extensively discussed inflammatory cells, we've identified indicators, such as those related to red blood cells and electrolytes, that have been less explored in prior research 28-30. These factors may significantly impact the prognosis of cancer patients, underscoring the need for further exploration in future studies.

To the best of our understanding, this study represented the most comprehensive effort to construct a prognosis model for patients with brain metastases using hematological parameters. The large cohort of this study ensured the robustness and reliability of the research findings. The utilization of parameters derived from routine blood tests enhanced the model's practical applicability in clinical settings. Methodological innovation based on RSF ensured the high flexibility and accuracy of the Model-GPAH. Furthermore, integrating Model-HP with the GPA model resulted in the formation of the new Model-GAPH, achieving further enhancement in model performance.

Several limitations should be considered. Firstly, the retrospective study design brings forth the potential for selection bias and residual confounding. Moreover, the patient cohort was recruited from a single institution, and the validation process was limited to internal validation. To affirm the model's widespread applicability, external validation is essential.

In summary, by applying machine learning methods, this study successfully identified the optimal hematological parameter model, designated as Model-HP, for patients with brain metastases. The integration of Model-HP into the traditional GPA risk model significantly improves the accuracy of prognosis predictions. Therefore, highlighting routine hematological test in patients with brain metastases is crucial for precise prognosis prediction and facilitating informed clinical and treatment decision-making. Future research should conduct external validation of Model-GAPH in multi-center, large-scale patient cohorts and carry out prospective studies to verify its clinical effectiveness. Additionally, other biomarkers and clinical indicators can be integrated to construct a more comprehensive prognostic assessment tool. Moreover, applying advanced algorithms such as deep learning can optimize model performance, while leveraging big data analysis to uncover prognostic-related factors. These efforts will provide effective support for prognostic assessment and clinical decision-making in patients with brain metastases.

## Supplementary Material

Supplementary figures and tables.

## Figures and Tables

**Table 1:** Characteristics of patients

Variables	Total (n = 1385)	Training cohort (n = 970)	Validation cohort (n = 415)	P-Value
Age (%)				0.156
<50	384 (27.7%)	254 (26.2%)	130 (31.3%)	
50-59	406 (29.3%)	293 (30.2%)	113 (27.2%)	
>60	556 (40.1%)	393 (40.5%)	163 (39.3%)	
Missing	39 (2.8%)	30 (3.1%)	9 (2.2%)	
Gender (%)				< 0.001
Male	816 (58.9%)	602 (62.1%)	214 (51.6%)	
Female	569 (41.1%)	368 (37.9%)	201 (48.4%)	
BMI (SD)	22.3 (3.2)	22.3 (3.3)	22.4 (3.1)	0.628
KPS (%)				0.316
<70	228 (16.5%)	151 (15.6%)	77 (18.6%)	
70-80	753 (54.4%)	538 (55.5%)	215 (51.8%)	
>80	390 (28.2%)	272 (28%)	118 (28.4%)	
Missing	14 (1%)	9 (0.9%)	5 (1.2%)	
Number of brain metastases (%)				0.586
1	478 (34.5%)	336 (34.6%)	142 (34.2%)	
2-3	142 (10.3%)	104 (10.7%)	38 (9.2%)	
>3	487 (35.2%)	335 (34.5%)	152 (36.6%)	
Missing	278 (20.1%)	195 (20.1%)	83 (20%)	
Primary cancer (%)				0.195
Lung cancer	1080 (78%)	753 (77.6%)	327 (78.8%)	
Nasopharyngeal carcinoma	86 (6.2%)	64 (6.6%)	22 (5.3%)	
Breast cancer	51 (3.7%)	30 (3.1%)	21 (5.1%)	
Other	168 (12.1%)	123 (12.7%)	45 (10.8%)	
Targeted therapy (%)				0.232
No	951 (68.7%)	676 (69.7%)	275 (66.3%)	
Yes	434 (31.3%)	294 (30.3%)	140 (33.7%)	
Radiotherapy (%)				0.267
No	711 (51.3%)	488 (50.3%)	223 (53.7%)	
Yes	674 (48.7%)	482 (49.7%)	192 (46.3%)	
Gamma knife radiosurgery (%)				0.185
No	1141 (82.4%)	790 (81.4%)	351 (84.6%)	
Yes	244 (17.6%)	180 (18.6%)	64 (15.4%)	
Chemotherapy (%)				0.394
No	499 (36%)	342 (35.3%)	157 (37.8%)	
Yes	886 (64%)	628 (64.7%)	258 (62.2%)	
Extracranial metastases (%)				0.851
No	484 (34.9%)	341 (35.2%)	143 (34.5%)	
Yes	901 (65.1%)	629 (64.8%)	272 (65.5%)	

## Abbreviations

GPA: graded prognostic assessment
KPS: karnofsky performance status
ML: machine learning
LASSO: least absolute shrinkage and selection operator
RSF: random survival forest
OS: overall survival
AIC: akaike information criterion
VIMP: variable importance
AUC: area under the receiver operating characteristic curve
cNRI: continuous net reclassification improvement
IDI: integrated discrimination improvement
SD: standard deviation
LDH: lactate dehydrogenase
AAPR: albumin-to-alkaline phosphatase ratio
FIB: fibrinogen
TT: thrombin time
HDL: high-density lipoprotein
