# Supplementary material for: Machine Learning-based Prognostic Model for Brain Metastasis Patients: Insights from Blood Test Analysis
Source: J Cancer. 2025 Jan 1;16(2):689–99. doi: 10.7150/jca.103847 (PMC11685702; doi:10.7150/jca.103847)

Table. S1 The included hematological parameters.

**Hematological parameters**	
Absolute Basophils	/
Absolute Eosinophils	/
Absolute Lymphocytes	/
Absolute Monocytes	/
Absolute Neutrophils	/
Albumin	/
ALP	Alkaline Phosphatase
ALT	Alanine Aminotransferase
Anion Gap	
Anti HCV Ab	Anti-Hepatitis C Virus Antibody
APTT	Activated Partial Thromboplastin Time
APTT Ratio	Activated Partial Thromboplastin Time Ratio
AST	Aspartate Aminotransferase
AST/ALT	Aspartate Aminotransferase / Alanine Aminotransferase
Basophil Percentage	/
Ca	Calcium
Cholesterol	Total Cholesterol
CK	Creatine Kinase
Cl	Chloride
CO2CP	Carbon Dioxide Content
Creatinine	/
Direct Bilirubin	/
Eosinophil Percentage	/
Fibrinogen	/
Globulin	/
Glucose	/
GT	Gamma-Glutamyl Transferase
Hb	Hemoglobin
HBDH	Lactate Dehydrogenase Isoenzyme 1
HCT	Hematocrit
HDL	High-Density Lipoprotein
Immature Granulocytes Absolute	/
Immature Granulocytes Percentage	/
Indirect Bilirubin	/
INR	International Normalized Ratio
K	Potassium
LDH	Lactate Dehydrogenase
LDL	Low-Density Lipoprotein
Lymphocyte Percentage	/
MCH	Mean Corpuscular Hemoglobin
MCHC	Mean Corpuscular Hemoglobin Concentration
MCV	Mean Corpuscular Volume
Mg	Magnesium
Monocyte Percentage	/
Na	Sodium
Neutrophil Percentage	/
NLR	Neutrophil-to-Lymphocyte Ratio
Platelet Count	/
PT	Prothrombin Time
PT Ratio	Prothrombin Time Ratio
RBC Count	Red Blood Cell Count
RDW-CV	Red Cell Distribution Width - Coefficient of Variation
RDW-SD	Red Cell Distribution Width - Standard Deviation
Serum Beta-Hydroxybuty rate	/
Serum Cystatin C	/
Serum P	Serum Phosphorus
Total Bilirubin	/
Total Protein	/
Triglycerides	/
TT	Thrombin Time
Urea	Blood Urea Nitrogen
Uric Acid	/
WBC Count	White Blood Cell Count

Table.S2 The significant hematological parameters in reference model. LDH，Lactate Dehydrogenase；GT，Gamma-Glutamyl Transferase；CK，Creatine Kinase.

Variables	HR	95% CI	P-value
Ca	2.795	(1.171, 6.668)	0.021
LDH	1.004	(1.000, 1.008)	0.045
GT	0.997	(0.995, 1.000)	0.021
CK	1.002	(1.000, 1.003)	0.007
Glucose	1.142	(1.051, 1.241)	0.002
Serum Beta-Hydroxybuty rate	2.064	(1.067, 3.991)	0.031
Platelet Count	0.998	(0.996, 1.000)	0.016
Immature Granulocytes Absolute	14.229	(2.072, 97.715)	0.007

Table.S3 The 1-year, 3-year and 5-year IDI and cNRI comparison among comprehensive model, Univariate Cox regression, Stepwise regression, LASSO regression, and RSF. IDI, Integrated Discrimination Improvement; cNRI, continuous Net Reclassification Improvement; LASSO, Least absolute shrinkage and selection operator; RSF, random survival forest.

Model comparison (RSF vs.)	1-year IDI comparison			3-year IDI comparison			5-year IDI comparison		
	Estimate	95% CI	P value	Estimate	95% CI	P value	Estimate	95% CI	P-value
Reference model	0.086	(0.040, 0.147)	0	0.148	(0.090, 0.195)	0	0.115	(0.050, 0.165)	0.01
Univariate Cox regression	0.070	(0.021.0.128)	0	0.135	(0.078, 0.175)	0	0.103	(0.054, 0.155)	0
Stepwise regression	0.096	(0.055, 0.151)	0	0.149	(0.095, 0.200)	0	0.114	(0.060, 0.169)	0
Lasso regression	0.080	(0.039, 0.128)	0	0.134	(0.087, 0.178)	0	0.102	(0.053, 0.153)	0

Model comparison (RSF vs.)	1-year cNRI comparison			3-year cNRI comparison			5-year cNRI comparison		
	Estimate	95% CI	P value	Estimate	95% CI	P value	Estimate	95% CI	P-value
Reference model	0.214	(0.127, 0.343)	0	0.386	(0.286, 0.476)	0	0.315	(0.074, 0.438)	0.01
Univariate Cox regression	0.217	(0.119, 0.344)	0	0.371	(0.244, 0.455)	0	0.339	(0.145,0.482)	0
Stepwise regression	0.282	(0.168, 0.377)	0	0.404	(0.261, 0.478)	0	0.349	(0.141, 0.486)	0
Lasso regression	0.263	(0.129,0.358)	0	0.166	(0.106, 0.217)	0	0.378	(0.156, 0.500)	0

Table.S4 Comparison of the 1-year, 3-year, and 5-year IDI, and cNRI among Model-HP, GPA, and Model-GPAH. GPA, Graded Prognostic Assessment; Model-HP, model based on hematological parameters; Model-GPAH, model based on GPA and hematological parameters.

Model comparison	1-year IDI comparison			3-year IDI comparison			5-year IDI comparison		
	Estimate	95% CI	P value	Estimate	95% CI	P value	Estimate	95% CI	P-value
Model-GPAH vs. Model-HP	0.012	(-0.002, 0.027)	0.070	0.011	(0.001, 0.026)	0.010	0.007	(-0.004, 0.022)	0.209
Model-GPAH vs. GPA	0.047	(0.025, 0.066)	0	0.046	(0.024, 0.063)	0	0.047	(0.028, 0.067)	0
Model-HP vs. GPA	0.035	(0.011, 0.058)	0.020	0.035	(0.008, 0.056)	0.010	0.039	(0.012, 0.064)	0

Model comparison	1-year cNRI comparison			3-year cNRI comparison			5-year cNRI comparison		
	Estimate	95% CI	P value	Estimate	95% CI	P value	Estimate	95% CI	P-value
Model-GPAH vs. Model-HP	0.118	(-0.025, 0.186)	0.090	0.107	(-0.008, 0.193)	0.070	0.029	(-0.096, 0.188)	0.567
Model-GPAH vs. GPA	0.206	(0.117, 0.263)	0	0.214	(0.132, 0.264)	0	0.262	(0.117, 0.335)	0
Model-HP vs. GPA	0.141	(0.042, 0.222)	0.010	0.146	(0.025, 0.215)	0.040	0.163	(0.022, 0.293)	0.020

Table. S5 The imputed baseline characteristics of included patients.

Variables	Total (n = 1385)	Training cohort (n = 970)	Validation cohort (n = 415)	P-Value
Age (%)				0.147
<50	392 (28.3%)	260 (26.8%)	132 (31.8%)	
50-59	409 (29.5%)	296 (30.5%)	113 (27.2%)	
>60	584 (42.2%)	414 (42.7%)	170 (41%)	
Gender (%)				< 0.001
Male	816 (58.9%)	602 (62.1%)	214 (51.6%)	
Female	569 (41.1%)	368 (37.9%)	201 (48.4%)	
BMI (SD)	22.4 (2.8)	22.3 (2.8)	22.4 (2.6)	0.616
KPS (%)				0.291
<70	229 (16.5%)	151 (15.6%)	78 (18.8%)	
70-80	761 (54.9%)	543 (56%)	218 (52.5%)	
>80	395 (28.5%)	276 (28.5%)	119 (28.7%)	
Number of brain metastases (%)				0.281
1	665 (48%)	474 (48.9%)	191 (46%)	
2-3	142 (10.3%)	104 (10.7%)	38 (9.2%)	
>3	578 (41.7%)	392 (40.4%)	186 (44.8%)	
Primary cancer (%)				0.195
Lung cancer	1080 (78%)	753 (77.6%)	327 (78.8%)	
Nasopharyngeal carcinoma	86 (6.2%)	64 (6.6%)	22 (5.3%)	
Breast cancer	51 (3.7%)	30 (3.1%)	21 (5.1%)	
Other	168 (12.1%)	123 (12.7%)	45 (10.8%)	
Target (%)				0.232
No	951 (68.7%)	676 (69.7%)	275 (66.3%)	
Yes	434 (31.3%)	294 (30.3%)	140 (33.7%)	
Radiotherapy (%)				0.267
No	711 (51.3%)	488 (50.3%)	223 (53.7%)	
Yes	674 (48.7%)	482 (49.7%)	192 (46.3%)	
Gamma knife radiosurgery (%)				0.185
No	1141 (82.4%)	790 (81.4%)	351 (84.6%)	
Yes	244 (17.6%)	180 (18.6%)	64 (15.4%)	
Chemotherapy (%)				0.394
No	499 (36%)	342 (35.3%)	157 (37.8%)	
Yes	886 (64%)	628 (64.7%)	258 (62.2%)	
Extracranial metastases (%)				0.851
No	484 (34.9%)	341 (35.2%)	143 (34.5%)	
Yes	901 (65.1%)	629 (64.8%)	272 (65.5%)	

Figure.S1 The forest plot of multivariable Cox analysis based on variables selected through univariate Cox regression.

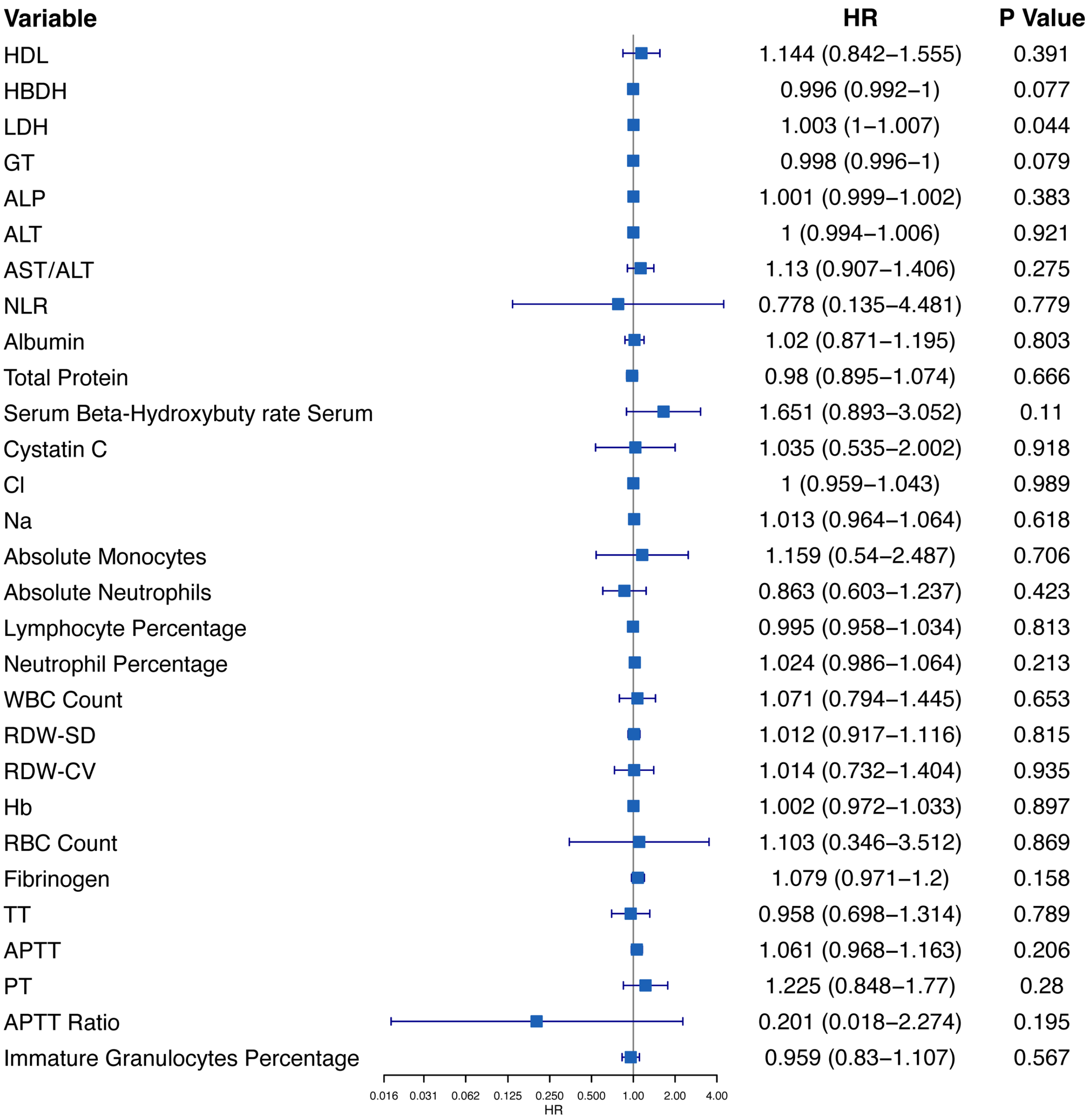

Figure.S2 The forest plot of multivariable Cox analysis based on variables selected through stepwise regression.

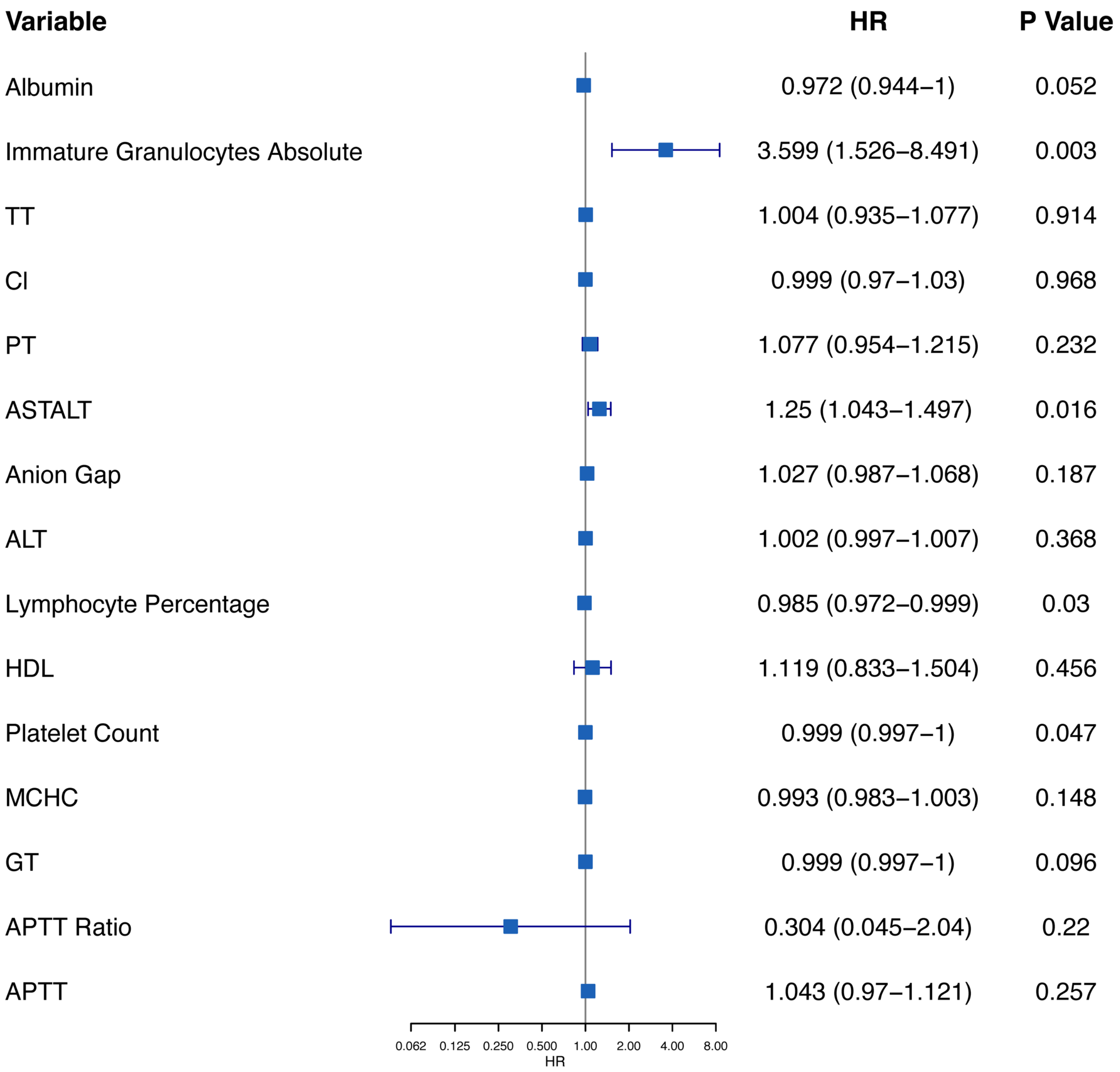

Figure.S3 Selection of significant hematological parameters in the training. (A) Cross-validation performed ten times to fine-tune parameter selection in the LASSO model. (B) Profiles of LASSO coefficients. LASSO, Least absolute shrinkage and selection operator. (C) The forest plot of multivariable Cox analysis based on variables selected through LASSO.

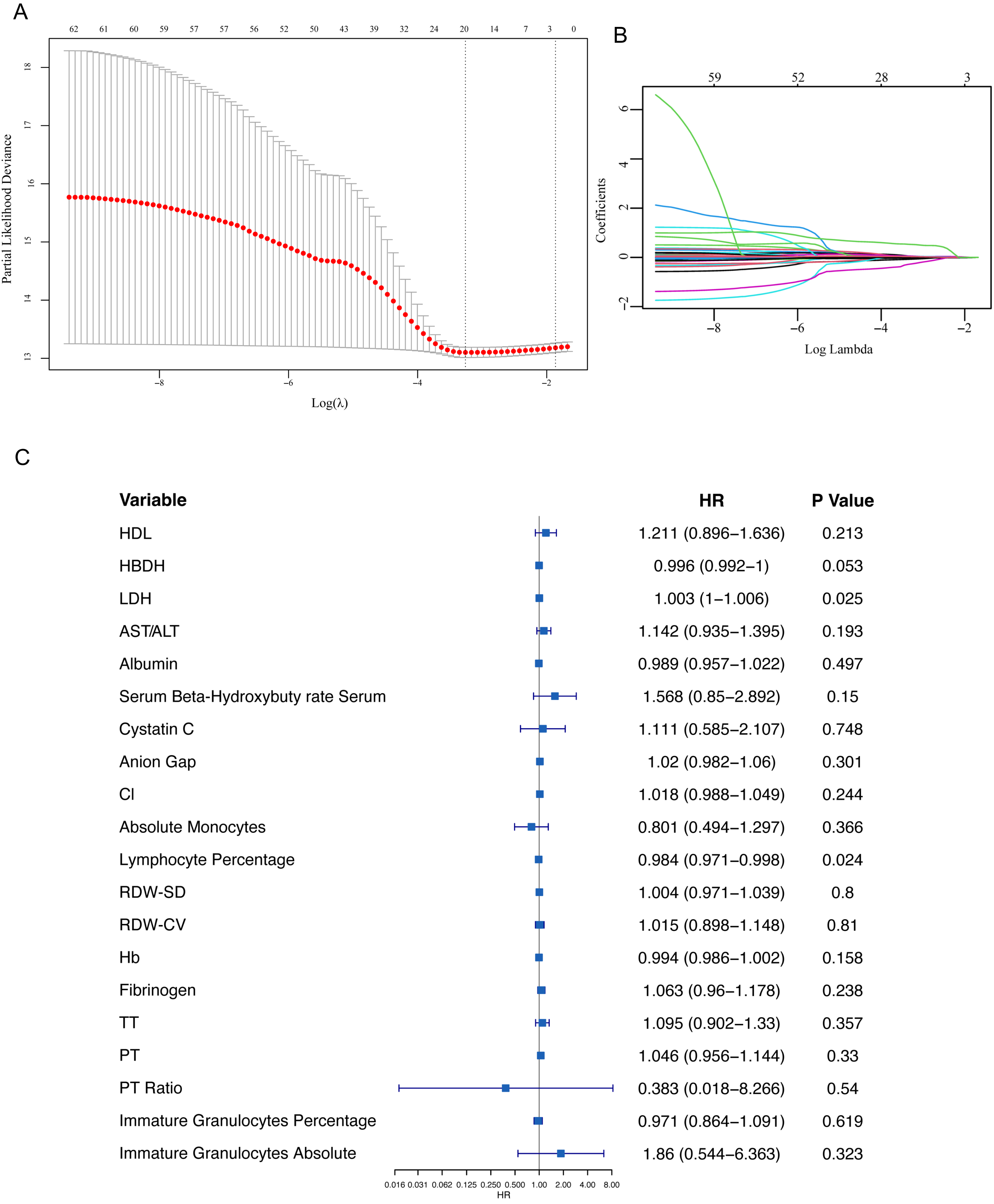

Figure.S4 Variable importance and error rate curve of RSF. RSF, random survival forest.

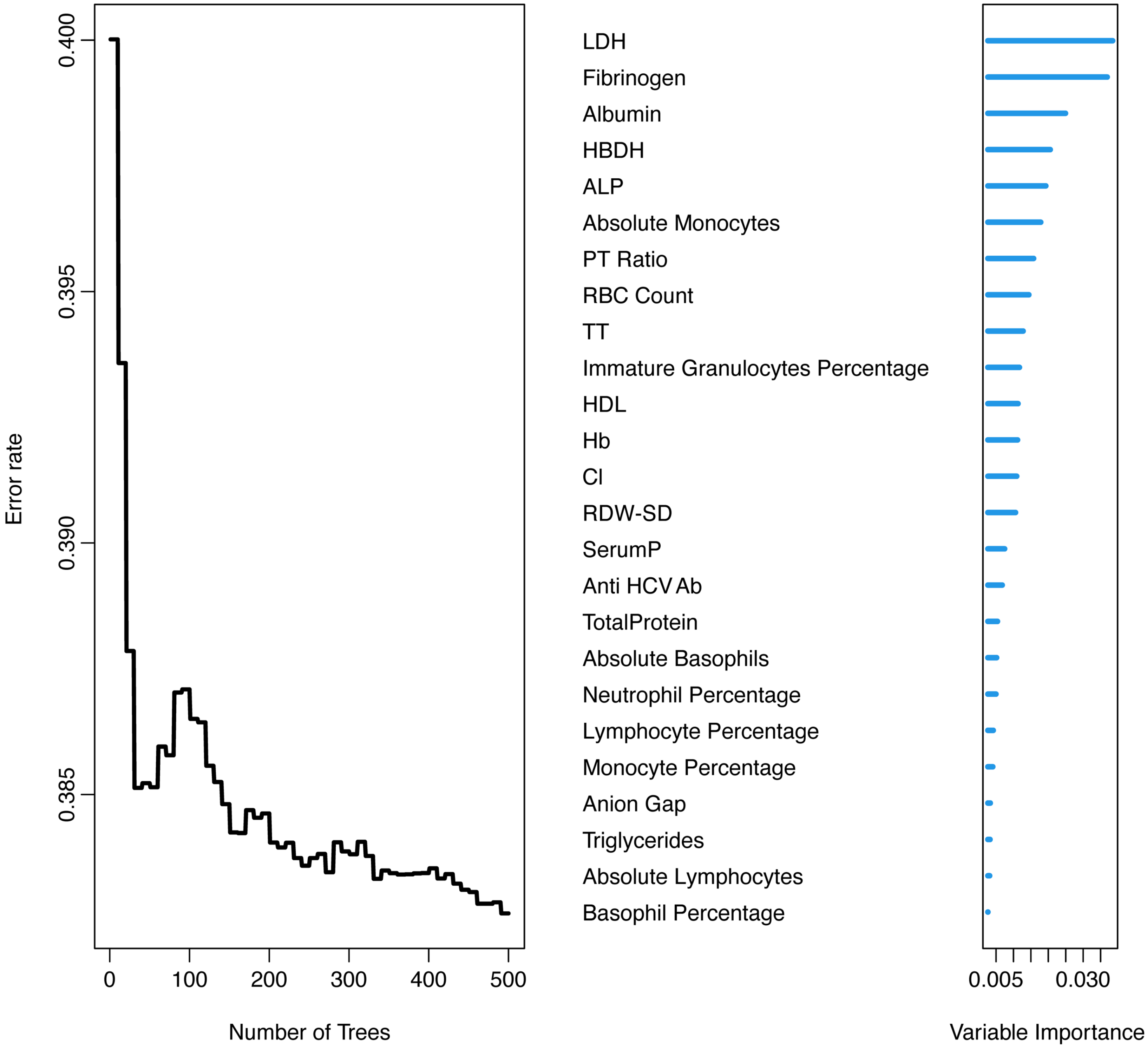

Figure.S5 The calibration plots of Model-GPAH.

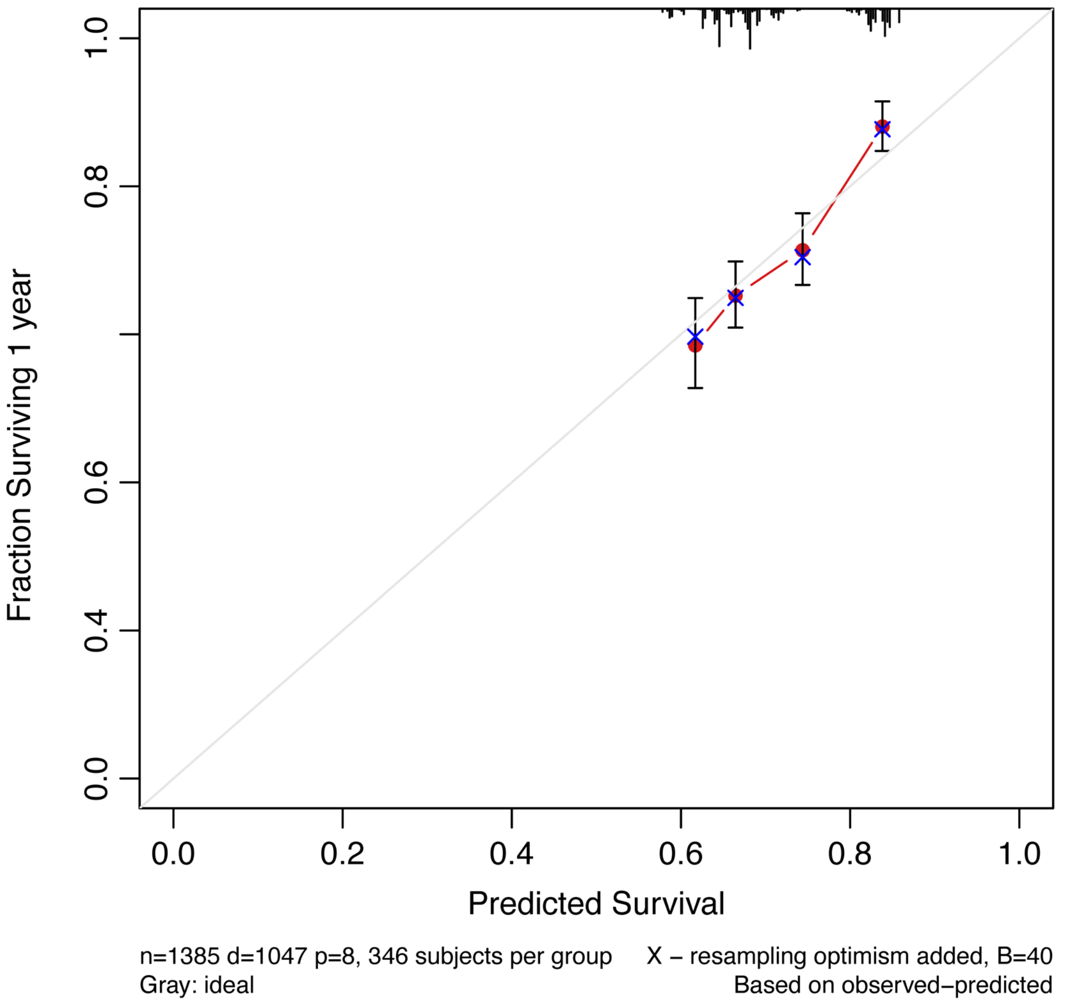

Figure.S6 Kaplan–Meier curves of OS for patients in different risk levels in the cohort without imputation. OS, overall survival.

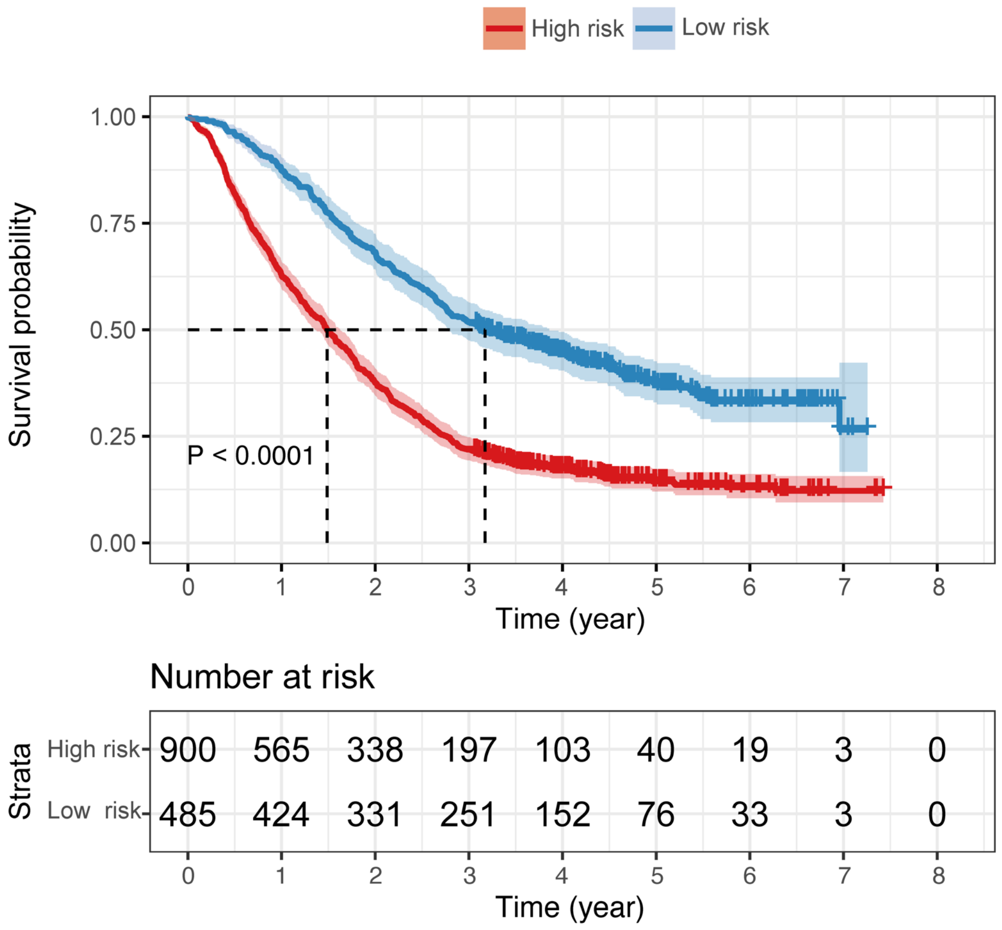

Supplement: Supplementary file 1 — Supplementary figures and tables. [file jcav16p0689s1.pdf]
